# Functional metagenomic profiling of intestinal microbiome in extreme ageing

**DOI:** 10.18632/aging.100623

**Published:** 2013-12-10

**Authors:** Simone Rampelli, Marco Candela, Silvia Turroni, Elena Biagi, Sebastiano Collino, Claudio Franceschi, Paul W O'Toole, Patrizia Brigidi

**Affiliations:** ^1^ Department of Pharmacy and Biotechnology, University of Bologna, Bologna, Italy; ^2^ Nestlé Institute of Health Sciences SA, Molecular Biomarkers, EPFL Innovation Park, bâtiment H, 1015 Lausanne, Switzerland; ^3^ Department of Experimental, Diagnostic and Specialty Medicine, St. Orsola-Malpighi University Hospital, Bologna, Italy; ^4^ School of Microbiology & Alimentary Pharmabiotic Centre, University College Cork, Ireland

**Keywords:** centenarians, extreme-aging, gut microbiome

## Abstract

Age-related alterations in human gut microbiota composition have been thoroughly described, but a detailed functional description of the intestinal bacterial coding capacity is still missing. In order to elucidate the contribution of the gut metagenome to the complex mosaic of human longevity, we applied shotgun sequencing to total fecal bacterial DNA in a selection of samples belonging to a well-characterized human ageing cohort. The age-related trajectory of the human gut microbiome was characterized by loss of genes for shortchain fatty acid production and an overall decrease in the saccharolytic potential, while proteolytic functions were more abundant than in the intestinal metagenome of younger adults. This altered functional profile was associated with a relevant enrichment in “pathobionts”, i.e. opportunistic pro-inflammatory bacteria generally present in the adult gut ecosystem in low numbers. Finally, as a signature for long life we identified 116 microbial genes that significantly correlated with ageing. Collectively, our data emphasize the relationship between intestinal bacteria and human metabolism, by detailing the modifications in the gut microbiota as a consequence of and/or promoter of the physiological changes occurring in the human host upon ageing.

## INTRODUCTION

Ageing is a complex multifactorial process with a major impact on the human body [1]. The ageing process seriously affects the human gut microbiota in particular, because it is accompanied by changes in the physiology of the gastrointestinal tract and associated immune system, as well as by changes in diet and lifestyle [2,3]. The adult-like profile of the human intestinal microbiota is stably maintained over time in healthy adults [4,5], defining an essentially mutualistic scenario whereby, in return for a guaranteed nutrient supply, the gut microbiota provides numerous fundamental functions to the host, including vitamin and metabolite supply and colonization resistance against pathogens [6]. The pathophysiology of the ageing process can ultimately compromise this homeostasis with a subject-specific timing, depending on the individual physiological status, diet, lifestyle and frailty [7-13].

Changes in microbiota composition in older people have been connected to immunosenescence and inflammaging [14-17]. An important factor which affects the gut microbiota in ageing is diet [18]. A very recent study highlighted the impact of the habitual diet on the gut microbiota of elderly people, demonstrating diet-driven alterations in varying rates of health decline upon ageing [19]. In that study, a reduced coding capacity for producing short-chain fatty acids in frail subjects correlated with lower levels of butyrate, acetate and propionate in the fecal metabolome. However, the functional aspects of the intestinal microbial community and their relation with the ageing process are not frequently investigated. Indeed, the majority of recent studies focussing on the relationship between ageing and the gut microbiota used a 16S rDNA amplicon sequencing approach, which reflects the phylogenetic structure of the microbiota but does not provide information pertaining to function. Even if microbiota compositional information allows a large number of deductions to be inferred, a metagenomics approach is ultimately required, because it provides a view of community structure in terms of species richness and distribution, as well as the functional (metabolic) potential of the community metagenome [20].

In order to explore the age-related changes of the human gut microbiome, we applied Illumina shotgun sequencing to 9 fecal samples, whose microbiota composition was reported in a previous study [8], including centenarians, individuals at the upper extremity of the human lifespan [21]. We hypothesized that centenarians represent a valuable opportunity for increasing the temporal resolution of microbiota-ageing interactions. The particular physiology and the general inflammatory status of centenarians make these individuals very different from the rest of the population, including in terms of gut microbiota composition [8]. In a very recent study, the metabolomic profile of centenarians was also characterized, allowing the identification of some urine metabotypes which were strongly connected with extreme ageing and some intestinal microbiota elements [22]. In the present pilot work, by determining the centenarian gut microbiome we propose a wide functional description of the human gut microbiota upon ageing.

## RESULTS

### Meta-analysis of gut microbiota functionality, taxonomy and urine metabotypes

We previously reported substantial inter-individual variability in the fecal microbiota composition of 64 older people, comprising 21 centenarians, 22 elderly subjects genetically unrelated to the centenarians, and 21 offspring of the centenarians [8]. A group of 20 young adults had also been included as a control. Furthermore, the metabolic phenotype of the subjects was recently characterized by ^1^H-NMR and mass spectrometry analysis of plasma and urine [22].

To investigate functional differences in the gut microbiome across the age groups studied and to characterize the metabolic trajectory of human intestinal microbiota upon ageing, we analyzed a subset of these samples: 3 centenarians, aged 99 to 102 years (mean 100.7), 5 elderly people, including 3 offspring of the centenarians, aged 59 to 75 years (mean 66.4), and one 38-year-old adult, as a young control. All the subjects were of Caucasian (Italian) ethnicity and resided in Emilia Romagna, a region in Northern Italy.

**Figure 1:**
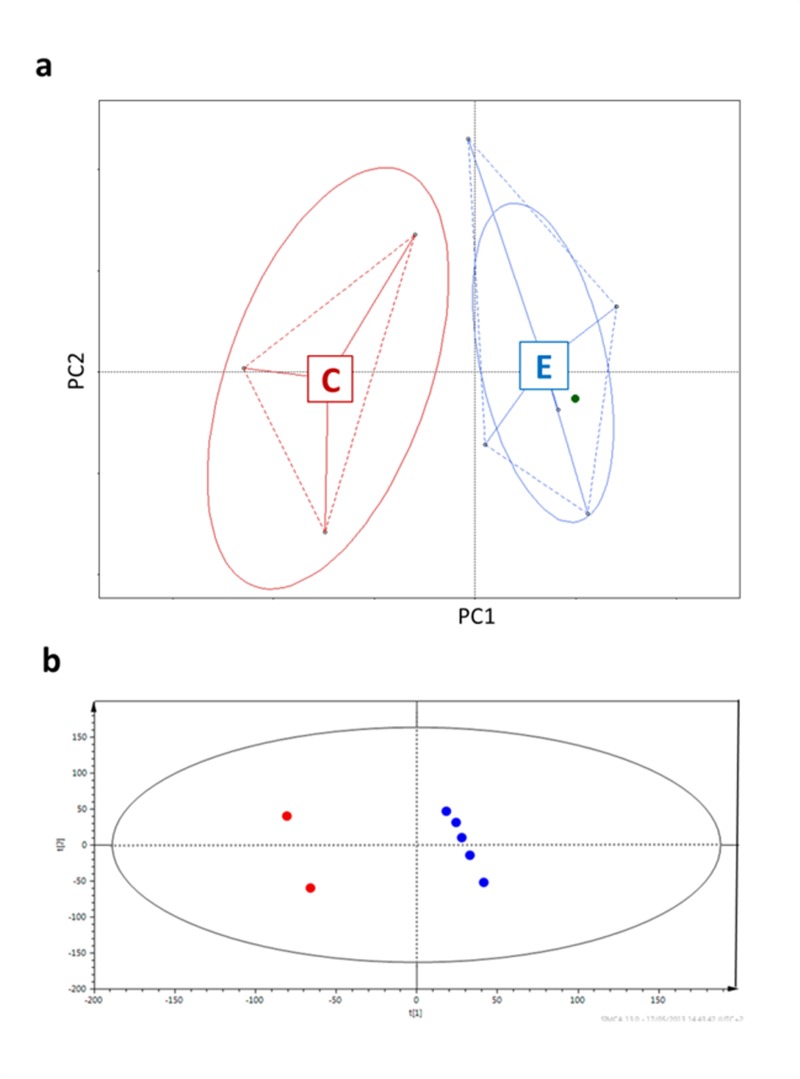
Centenarians and elderly differ in both microbiota composition (a) and urine metabotypes (b) **a**, Principal coordinates analysis (PCoa) of Euclidean distances between HITChip profiles [8]. PC1 represents 36.46% of the variability, PC2 19.91%. **b**, Two centenarian subjects versus elderly group; R^2^ = 0.394, Q^2^ = 0.596, two-component model. The ellipses represent the Hotelling's T2 with 95% confidence. Subjects are colored according to the age groups: red for centenarians, blue for elderly people, green for the young adult.

Firstly, in order to prove that the selected samples were representatives of the entire cohort they were drawn from, we performed a multivariate analysis on the previous HITChip results for the subjects selected for this study [8] (Figure 1a) and described their urine metabolic profiles applying Projection on Latent Structures – Discriminant Analysis (PLS-DA) on the ^1^H-NMR spectra [22] (Figure 1b). Both analyses showed that centenarians widely differed from the other subjects.

**Figure 2:**
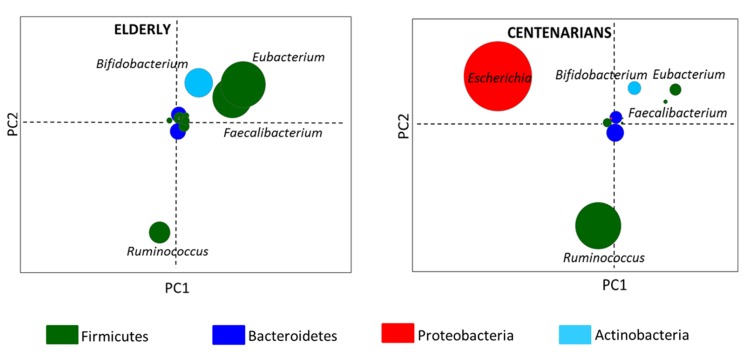
Taxonomic fingerprint of ageing Genus-node fingerprints were obtained using Euclidean PCoA. Each circle represents a bacterial genus colored on the basis of the phylum classification. Circle diameter is proportional to the average relative abundance of the bacterial genus in the corresponding age group.

By Illumina shotgun sequencing of the fecal microbial DNA from the selected individuals, we generated a total of 214.6 million paired-end reads, with an average of 23.841 million (± 0.067 SD) reads per subject. Shotgun sequences were processed using the MetaPhlAn pipeline and a multivariate analysis of the taxonomic results was carried out (Figure S1). By using the genus coordinates of the taxonomic PCoA and their mean contribution in the groups, two node graphs, respectively for centenarians and 70-year-old subjects, were obtained. Figure 2 thus shows the gut microbiota fingerprints at the genus level in centenarians and elderly. In the center of the resulting graphs were positioned the genus-nodes that were equally abundant in all the samples while the other areas of the graph contained genus-nodes present at different relative abundance across the subjects. Thus the data indicate that the genera *Escherichia* and *Ruminococcus* were over-represented in centenarians, whereas *Faecalibacterium*, *Eubacterium* and *Bifidobacterium* were more abundant in the elderly.

**Figure 3:**
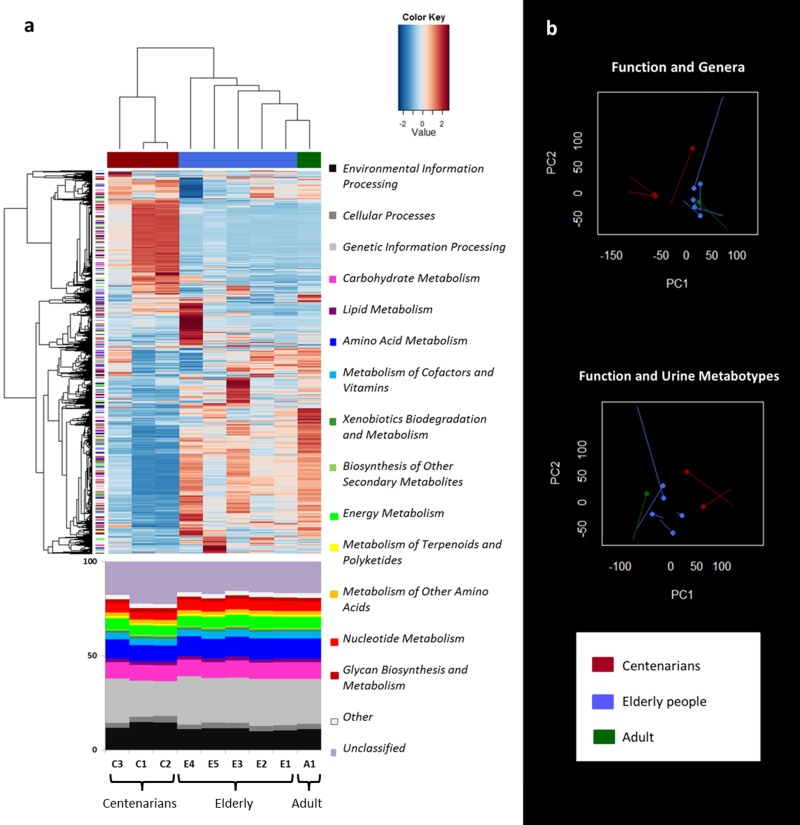
Metagenome function analysis separates centenarians from the other subjects in agreement with genus and urine metabolite clustering **a**, Hierarchical Ward-linkage clustering based on the Pearson correlation coefficients of the abundance of KO genes, filtered for KO gene subject presence ≥ 1 in at least 8/9 subjects. KO genes are clustered in the vertical tree and color-coded according to the first level of KEGG classification or the second level for functions concerning metabolism. 2719 KO genes confidently classified in the KEGG database are visualized. The bottom panel shows the relative abundance of the KEGG categories. **b**, Procrustes analysis combining Euclidean PCoA of functional microbiota (non-circle end of lines) with either Euclidean PCoA based on the genus dataset (circle-end of lines; upper graph) [8], or Euclidean PCoA based on the spectra of urine metabolites (circle-end of lines; lower graph) [22]. In both graphs color codes are per age group as in Figure 1.

Functional characterization of the shotgun sequence reads in the KEGG database was carried out by using MetaCV pipeline. Hierarchical Ward-linkage clustering based on the Pearson correlation of the normalized number of mapped reads per gene function at KEGG Orthology (KO) level showed a clear separation between centenarians (C1-C3) and the other subjects (E1-E5 and A1; Figure 3a). When examining the functional gene distribution at high levels of the KEGG database, we identified clusters of specific genes which were characteristic of the ageing groups, even if the relative abundance of functions was distributed in a similar way across all subjects (Figure 3a).

Procrustes analysis of the functional gene profiles and the microbiota β-diversity or Euclidean distances of urine metabotypes was used to co-illustrate the data (Figure 3b). In both cases, the separation between centenarians and the other subjects occurred along the first axis. The RV coefficient obtained by co-inertia analysis between the same paired datasets, highlighted the association between the taxonomic and functional datasets (RV = 0.794), in despite of the functional and urine metabonomic results, which appeared to be not related to each other (RV = 0.380).

### Relation between the functional structure of the gut microbiota and ageing

**Figure 4:**
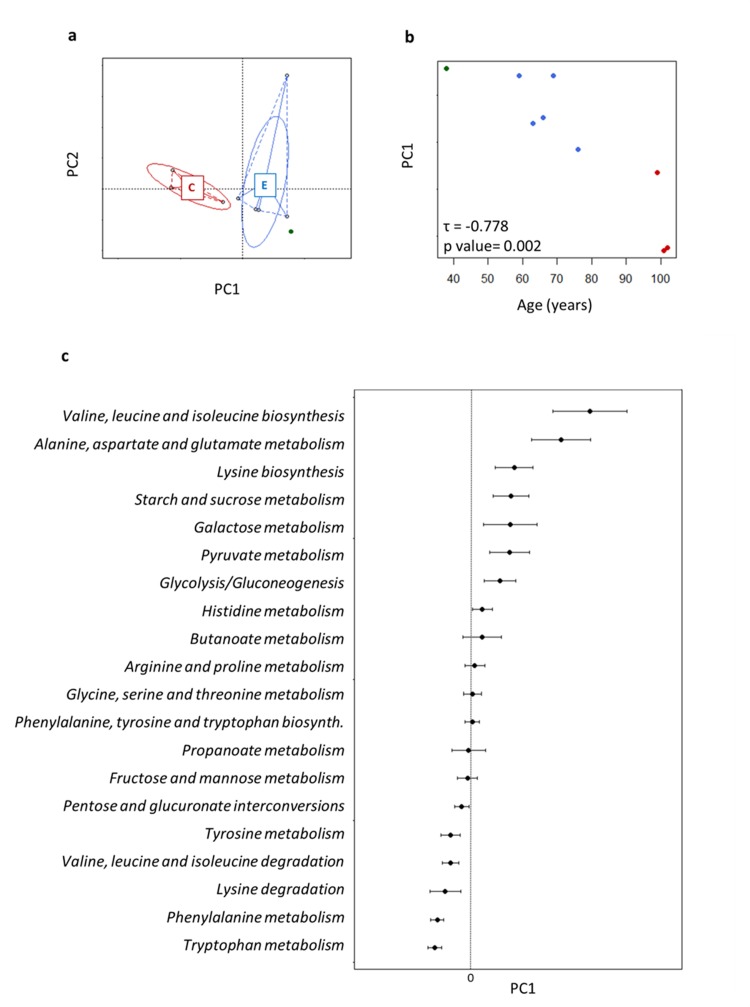
Age-related trajectory of gut microbiome functions **a**, PCoA of Euclidean distances between KEGG KO profiles. Subjects are colored as in Figure 1. **b**, The position of each fecal microbiome along PC1, which described the largest amount of variation (82.5%) in the dataset, was plotted against age. Each circle is a subject colored as in Figure 1. PC1 was significantly related with age. **c**, Average (± SEM, error bar) PC1 coordinates obtained for functional cluster of KO genes, corresponding to KEGG pathways, used for building the PCoA. In this way pathways are ordered for: (1) negative value of PC1 coinciding with high concordance with ageing profile; (2) value of PC1 close to 0 coinciding with constant presence in the dataset; (3) positive value of PC1 coinciding with high concordance with the healthy adult profile.

We applied the metaCV procedure for achieving a functional characterization of the fecal microbiota at different levels of KEGG classification. Alpha-diversity was computed at KO level using the Simpson index and was not significantly different between the age groups (centenarians, 0.904 ± 0.010; the elderly, 0.892 ± 0.061). PCoA based on Euclidean distances of functional gene profiles at KO level showed an age-related resolution (Figure 4a). In particular, the first axis, which represented 82.5% of the data variability, described most of the ageing-related differences (Kendall's tau coefficient = −0.778, p value = 0.002, Figure 4b). For this reason, we used PCo1 to assess the functional potential of the gut microbiome upon ageing. In particular, we focused our attention on the genes involved in amino acid and carbohydrate metabolisms, core genes in metabolism, in order to obtain an ordination of the pathways along PCo1. Each point of the resulting graph corresponded to the average of the coordinates of the KO genes involved in the same KEGG pathway, and the intervals coincided with the standard error of the mean (SEM; Figure 4c). In this way, we built a sort of ranking of KEGG pathways, where negative values of PCo1 coordinates were related with ageing. We observed a polarization of the pathways related to amino acid metabolism in the extremities of the axis, and a more dense localization at positive values of PCo1 coordinates for the pathways involved in carbohydrate metabolism. In particular, the metabolism of two aromatic amino acids, tryptophan and phenylalanine, was closely associated with ageing, followed by the metabolism of other amino acids, such as tyrosine, valine and lysine. Conversely, the metabolism of other amino acids and several carbohydrates, such as glucose, galactose, histidine, pyruvate and butanoate was related to high positive values of PCo1. Notably, the last two pathways are strictly connected with short-chain fatty acid (SCFA) production.

### Functional signature of extreme ageing in the intestinal core microbiome

Since we aimed at highlighting alterations within the core microbiome structure associated with ageing, we first filtered out all the KO genes which possessed a significant (p value < 0.05) positive correlation with the proportional abundance of *Escherichia*, a pathobiont that is prevalent in the centenarian gut. The filtered genes were further analyzed for their correlation with subject age (p value < 0.05). The resulting 116 KO genes are listed in Supplementary Table 1, with annotated functions.

## DISCUSSION

In the present study we characterized the gut microbiota metagenome of extreme longevity individuals, elderly people and a young adult by Illumina shotgun sequencing. The subjects chosen typify a bigger cohort whose members had been well characterized for gut microbiota composition as well as for urine and serum metabolites[8, 22]. Here we focused on the functional aspects of the gut microbiota, in order to investigate ageing-related changes of the microbiome structure and function. Confirming Biagi et al. (2010) [8], multivariate analysis of the taxonomic dataset showed an overall increase in *Proteobacteria* and a re-arrangement in *Firmicutes* in centenarians, whereas 70-year-old elderly people maintained a gut microbiota profile very similar to the one shown by the young adult. The Procrustes analysis highlighted the relationships between taxonomic and functional datasets, showing a clear separation between centenarians and younger subjects with respect to both microbiome structure and functionality. Shotgun sequencing analysis revealed important modifications in microbiota functions in centenarians, allowing us to define an aged-type microbiome characterized by a specific functional complement different from the one present in younger subjects. As a result of taxonomic rearrangements in the gut microbiota composition, the changes in microbiome structure we detected as a function of aging allowed us to shed some light on the mechanisms associated with bacterial community change accompanying the overall ageing process.

Examining the KO gene abundance we identified a cluster including all the centenarian subjects, separated from the clustered younger subjects. While at high level of KEGG assignments the functional structure of the microbiota was present in both clusters without significant differences, the distribution of genes at KO levels followed an age-related tendency. Among these, we observed an age-related increased abundance of genes involved in the tryptophan metabolism pathway (ko00380). This evidence is in agreement with the reduction of tryptophan found in serum of centenarians [22], although we cannot directly infer causality. Linking together the two observations, we advance the hypothesis that the potential increase of consumption of tryptophan by the gut microbiota affects its bioavailability within the host. A recent study showed patients with inflammatory diseases to have a significant depletion of serum levels of tryptophan compared to control population [23] and Huang et al. demonstrated a clear relationship between reduced serum tryptophan levels and an increase of immune activation [24]. In addition, the decrease of the serum level of tryptophan was associated with cognitive deficit in senile dementias [25-27], and Noristani et al. demonstrated that high triptophan diet lead to a reduction of the plaque pathology in Alzheimer's disease in mouse [28]. It is thus tempting to speculate that a microbiota-dependent reduction of tryptophan can nurture inflammaging in centenarians and could worsen the conditions of the patients affected by cognitive deficit. Furthermore, our study revealed that the altered abundance of genes involved in phenylalanine metabolism (ko00360) and tyrosine metabolism (ko00350) was related with ageing. This evidence supports the proposal of two metabolites (phenylacetylglutamine (PAG) and p-cresol-sulfate (PCS)), as markers in urine of extreme ageing [22]. Indeed, it has been extensively demonstrated that PAG and PCS derive from microbial metabolism of aromatic amino acids, including tyrosine and phenylalanine [29]. Short-chain fatty acids – mainly acetate, butyrate and proprionate – are microbiota-derived metabolites that are fundamental for the human health and wellbeing. They represent a source of energy for the gastrointestinal epithelium, stimulators for the release of mucins, immune modulators, and promoters of the integrity of the epithelial barrier [30,31]. Primarily, SCFAs are produced by bacterial fermentation of dietary polysaccharides which reach the colon undigested. This process relies on the presence of a glycophilic bacterial community in the colon, and also on other bacterial groups acting in concert with these primary degraders [19,32,33]. It has been demonstrated that a reduced dietary intake of carbohydrates causes decreased numbers of butyrate and butyrate-producing bacteria such as *Roseburia* and *Eubacterium* [34], highlighting the importance of carbohydrate availability in the large intestine for the maintenance of a fibrolytic SCFA-producing microbial community. In relation to this, our data showed a loss of genes involved in SCFA production and an overall decrease in the saccharolytic potential as a function of aging. In particular, in the extremely aged-type microbiome we observed a decrease in starch and sucrose metabolism (ko00500), pyruvate metabolism (ko00620), galactose metabolism (ko00052) and glycolysis/gluconeogenesis (ko00010), accompanied by a concomitant loss of fibrolytic microorganisms belonging to the *Eubacterium*, *Bifidobacterium* and *Faecalibacterium* genera. Notably, pyruvate and butanoate metabolism, which showed an inverse association with aging, are described in KEGG database as pathways containing genes involved in SCFA production. Our data showed an overall rearrangement in the aged-type microbiome of the pathways involved in the production of SCFA via proteolytic fermentation. In particular, genes involved in lysine degradation (ko00310), implicated in butyrate and acetate production, were more abundant in centenarians, while elderly and adults were enriched in genes responsible for the metabolism of glutamate, aspartate and alanine (ko00250), which in turn are involved in the production of acetate, butyrate and propionate, respectively [35]. Nevertheless, the aged-type microbiome was characterized by an overall increase in proteolytic functions, as suggested also by the degradation of the branched-chain amino acids, valine, leucine and isoleucine (ko00280), which was strictly connected with the microbial metagenome of centenarians. Notably, the first catabolic intermediates of valine, isoleucine and leucine are the precursors of the branched-chain fatty acids that are involved in the cell membrane biosynthesis of inflammation-promoting Gram-negative bacteria [36,37]. Interestingly, the increased abundance of proteolytic functions in the aged-type microbiome is accompanied by lower lysine, valine, leucine and isoleucine biosynthetic capability (ko00290; ko00300). All these molecules are essential amino acids, with the respective biosynthetic pathways completely lacking in mammals. In particular, lysine plays an essential role in many cellular process, including the functionality of the pyruvate dehydrogenase complex [38,39]. Reduction in the microbiota production of essential amino acids as part of aging could thus have a primary role in compromising the overall nutritional state of elderly subjects, leading to sarcopenia.

In summary the proteolytic potential of the gut microbiota appeared to be enhanced upon ageing, even if there are changes of substrate, and a clear loss of genes involved in the metabolism of carbohydrates in the aged-type microbiota was observed. Furthermore, we found an age-related reduction of the abundance of genes in pathways involved in SCFA production. Based on differential abundance, the aged-type microbiota is structurally and functionally compromised, moving from a saccharolytic to a putrefactive metabolism. It is noteworthy that these microbiome alterations concord with the enrichment of genes belonging to pathobionts. Pathobionts are minor microbiota opportunists able to thrive in inflamed conditions, sustaining and nurturing the physiological inflammation [14]. We therefore postulate that pathobiont overgrowth can nurture a sort of pro-inflammatory loop which could worsen the health status of aged people. We can also hypothesize that in centenarians –the extreme limit of human life span – some readjustments take place within the core mutualistic microbiome functions that serve to offset detrimental activities associated with pathobiont overgrowth. In order to shed some light on this, we focused our analysis on the core microbiome changes occurring in centenarians. According to our findings, 116 genes usually present in different metagenome project databases [40-43], reflect the rearrangement of the core metabolic potential of the centenarian intestinal microbial ecosystem. This raises the question of whether these specific changes in the structure of the mutualistic counterpart of the gut microbiome reflect a new mutualistic configuration, responding to a microbiota-host adaptation process co-evolved to support the extreme limits of human lifespan.

It is important to note that in this pilot study the functional characterization of a small number of samples, representative of a bigger cohort, allowed us to investigate the functional potential of the gut microbiota, which clearly needs further validation by increasing the sample number. Furthermore, since the subjects belong to a limited geographic area (Emilia Romagna region), and are thus similar in lifestyle and habitual dietary patterns, these data also need confirmation across populations with different environmental conditions and genetic background. Nevertheless, our microbiome study of extreme ageing contributes to assess a more inclusive portrayal of the role of the intestinal bacterial counterpart in the pathophysiology of the ageing process. An ongoing project (www.nu-age.eu) will also include analysis of different dietary regimes in the ageing human population, to allow searching for correlations between gut microbiota composition and functionality, physiological and immunological parameters, metabonomic profiles and diet.

## METHODS

### Sample collection

The study employed 9 fecal samples collected for the study of Biagi et al. (2010) [8]. We utilized the same fecal genomic DNA used for previous analysis and which had been stored at −20°C.

### Basic bioinformatic and statistical analysis of the metadata

Phylogenetic profiles of HITChip array data were retrieved from the study of Biagi et al. (2010) [8]. The dataset was analyzed in R version 3.0.0 (www.cran.org), using the R package “Vegan” [44], in order to obtain a Principal Coordinates Analysis (PCoA) based on Euclidean distances.

Urine metabotypes of 8 out of 9 individuals involved in our study (2 centenarians, 5 elderly people and 1 adult) had been previously characterized by ^1^H-NMR profiling NMR(Bruker Avance III 600 MHz, Bruker Biospin, Rheinstetten, Germany) [22]. The resulting spectra were analyzed in the present study using the packages “ChemoSpec” [45] and “Vegan” [44]. Projection on Latent Structures – Discriminant Analysis (PLS-DA) plot was carried out with SIMCA software (version 13.0, Umetrics AB, Umea, Sweden).

The phylogenetic characterization of the shotgun sequences was achieved at different levels of taxonomy by using MetaPhlAn with default parameters [46]. Statistical analysis was carried out using R. MetaPhlan result files were read into R, and matrices containing the relative abundances of taxa at phylum and genus level for each sample were constructed. Abundance analysis, multivariate statistical analysis, including PCoA, were computed using Cytoscape [47], R Bioconductor package “Made4” [48], and the R packages “stats” and “Vegan” [44].

Integration of the data by Procrustes and Co-inertia analyses was achieved using the functions “procrustes” and “cia” of “Vegan” [44] and “Made4” [48] packages, respectively. The coefficient RV of Co-inertia Analysis describes the global similarity between datasets: the closer this value is to 1, the greater is the similarity.

### Gut microbiome functional characterization: bioinformatic and statistical analysis

In order to functionally annotate the data, the shotgun reads first had to be quality filtered to remove low quality and human sequences. This step was achieved by using the human sequence removal pipeline and the WGS read processing procedure of the Human Microbiome Project (HMP) [41]. The obtained reads were locally assigned for functionality at different levels of the KEGG database [49], using the Metagenome Composition Vector (MetaCV), with default parameters. This tool consists of a composition algorithm which allows classification of very short reads into functional groups [50]. The resulting table consists of a matrix, with sample IDs in the columns and annotations at different levels of KEGG database in the rows. The file produced was loaded into R and then filtered for the abundances at first and then for KEGG Orthology (KO) level of KEGG classification.

PCoA using Euclidean distances based on KO gene dataset was performed with the package “Vegan” of R [44] and used also for Procrustes analysis. The function “scores” of “Vegan” was used to retrieve the coordinates of the samples and KEGG KO genes in the PCoA. The values obtained were used to perform observations about their ordination along the axes. Kendall correlation test was achieved using function “cor.test” of the package “stats” of R. When appropriate, *p* values were adjusted for multiple comparison using the Benjamini-Hochberg correction. Sequences were also assembled using meta-velvet [51] and the resulting contigs were uploaded in MG-RAST [52] under the project ID 2558.

## SUPPORTING INFORMATION


