# Supplementary material for: Functional metagenomic profiling of intestinal microbiome in extreme ageing
Source: Aging (Albany NY). 2013 Dec 10;5(12):902–12. doi: 10.18632/aging.100623 (PMC3883706; doi:10.18632/aging.100623)
Supplement: Supplementary file 1 [file aging-05-902-s001.pdf]

SUPPORTING INFORMATION

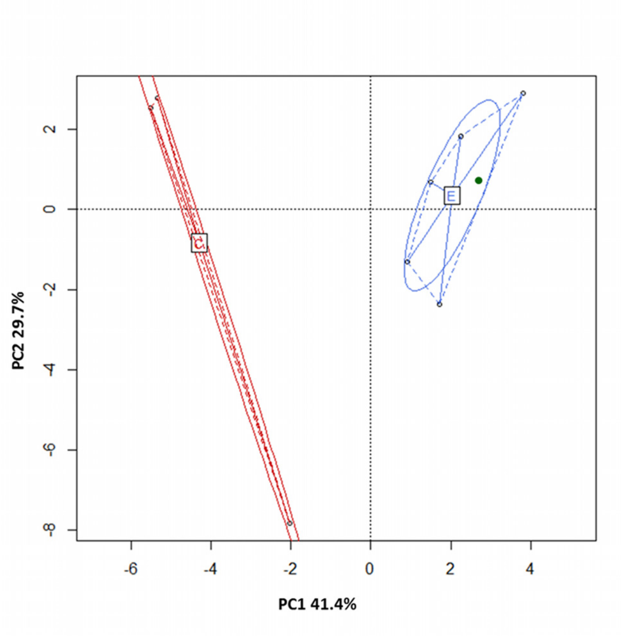

Figure S1. Centenarians and elderly subjects differ for gut microbiota composition. PCoA of Euclidean distances between genus-level taxonomic profiles. Subjects are colored as in Figure 1.

Please browse the full text versiom of this manuscript to see the Supplementary Table "KEGG Orthologous (KO) genes of core microbiome which correlated with ageing".
